# Effect of a positive Sea Surface Temperature anomaly on a Mediterranean tornadic supercell

**DOI:** 10.1038/s41598-017-13170-0

**Published:** 2017-10-09

**Authors:** Mario Marcello Miglietta, Jordi Mazon, Vincenzo Motola, Antonello Pasini

**Affiliations:** 1 Institute of Atmospheric Sciences and Climate, National Research Council (ISAC-CNR), Lecce, Italy; 2 Department of Physics. Universitat Politècnica de Catalunya–BarcelonaTech, Barcelona, Spain; 3 National Agency for new technologies, energy and sustainable economic development (ENEA), Ispra, Italy; 4 Institute of Atmospheric Pollution Research, National Research Council (IIA-CNR), Rome, Italy

## Abstract

Extreme events represent a topic of paramount importance and a challenge for modelling investigations. Due to the need of high-resolution models, the study of severe localized convective phenomena is even more critical, especially in relation to changes in forcing factors, such as sea surface temperatures (SSTs), in future climate scenarios. Here, we analyze the effect of changes in SSTs on the intensity of a tornadic supercell in the Mediterranean through modelling investigations. We show dramatic (nonlinear) changes for updraft helicity and vertical velocity, which measure the intensity of the supercell, even for variations of SST only of + /−1 K.

## Introduction

Scientific literature has recently shown that SST can significantly affect high impact weather. Numerical simulations proved that hurricane Sandy in 2012 would have dramatically intensified over a warmer SST^1^. Starting from the analysis of windstorm Xynthia, it was suggested that a warmer SST and potentially enhanced latent heat release in future climate conditions may increase the windstorm risk in southwestern Europe^2^. The increase in SST in the last 30 years was shown to be crucial in the July 2012 precipitation extreme near the Black Sea^3^.

The Mediterranean basin is regularly affected by severe convective events, often of limited predictability^4^, which are frequently related to the cyclone activity in the region^5,6^. Intense sea surface fluxes favor heavy rainfall especially in late Summer and in Fall^7^. The intensification and persistence of a tropical-like cyclone in the Mediterranean Sea were shown to depend significantly on SST^8^. Small scale SST features played a key role in some heavy rainfall events in Northern Italy^9^, while SST in specific sub-areas controlled the development of three precipitation episodes in Spain^10^. On the other hand, the effect of the Adriatic SST varied considerably in six case studies of intense precipitation over Italy^11^.

Minor attention is currently paid to the influence of SST on severe localized convective features, such as supercells and tornadoes; such studies mainly focused on the effect of El Niño Southern Oscillation on tornado activity in USA. In particular, the warm (cold) SST anomalies in the tropical Pacific associated with El Niño (La Niña) were shown to reduce (increase) the number of tornadoes over the central US^12^. Also, warm SST anomalies in the Gulf of Mexico, which are negatively correlated with the tropical Pacific SST, are a potential predictor for moist instability, by enhancing the low-level moisture transport, influencing the storm characteristics, and increasing the number of tornadoes in the US Central Plains in Spring^13–15^. Springtime SST anomalies in the North Atlantic were also found to be related to extreme US tornado outbreaks^16^. Moving to other regions, high values of SST were proposed to favor the development of strong tornadoes in Bangladesh^17^, and were observed in a recent tornado outbreak in South Australia^18^.

The influence of SST on severe convective events is a relevant issue for several reasons: on the one hand, understanding how occasional warm SST anomalies affect their potential development and intensification can help forecasters to better predict their occurrence; on the other hand, the limited resolution of SST analyses used as lower boundary condition in current numerical models (generally few km), raises the question of how small-scale SST perturbations affect the simulation of these events and may limit their predictability.

Moreover, the issue is interesting from a climate change perspective, considering the predicted increase of SST in several basins and the intrinsic difficulties of determining the changes in intensity, frequency, and location of tornadoes and supercells in future climate scenarios. Such difficulties depend on the very high resolution required for their proper representation, which is far from that available in the current climate simulations^19^, and on the deficiency of tornado datasets in most of the world. Thus, at present, the best one can do is to analyze the change in the parameters favorable to severe convection, obtained from downscaled high-resolution simulations nested into global circulation model projections. This category of studies has generally predicted an increase in the frequency of these events in future scenarios, associated with greater potential instability that offsets the predicted reduction in deep-layer shear, resulting in environments more favorable for severe thunderstorms^20–23^.

However, the latter approach provides only a rough indication of the expected changes of severe convection in regions with complex morphology, like the Mediterranean. In these areas, the location and intensity of severe convective weather depend decisively on meso-γ features, which can be properly simulated only using grid spacing of 1–2 km^24^. For example, high-resolution numerical simulations were necessary to identify the crucial role of the circulations induced by small-scale terrain features for the development of a tornadic event in Spain^25^, to show that mesoscale patterns controlled the evolution of a supercell in northeastern Italy^26^, and that the presence of steep mountains may represent an important factor for tornadogenesis in Greece^27^. Thus, the conditions of development in the Mediterranean are different from the more homogeneous, synoptic-scale setting typical of the US Great Plains^26^.

Considering also that the most comprehensive database available in Europe^28^ suffers from relevant gaps in the southern regions, only partially mitigated in the last few years^29,30^, one can understand that a classical “climatological” approach would not work properly to identify climatic changes in severe convection over the Mediterranean. Thus, for the points discussed above, the sensitivity analysis to SST even in a single case study can be of some interest for both its meteorological and climatic implications.

In the present study, we started from the simulation of a supercell spawning a tornado in the Mediterranean^31^. Since the simulation was able to properly reproduce the tracking and timing of the cell, one can better understand the mechanisms responsible for its triggering and development by undertaking some sensitivity tests with modified control parameters. In particular, additional simulations are performed with modified SST.

## Results

### Case study

The tornado originated as a waterspout over the Ionian Sea, made landfall in the port of Taranto (Apulia region, southeastern Italy) after about 30 minutes^31^ (0950 UTC, 1050 Local Time, 28 November 2012), and was responsible for one fatality and estimated damages for 60 M€^32^.

**Figure 1:**
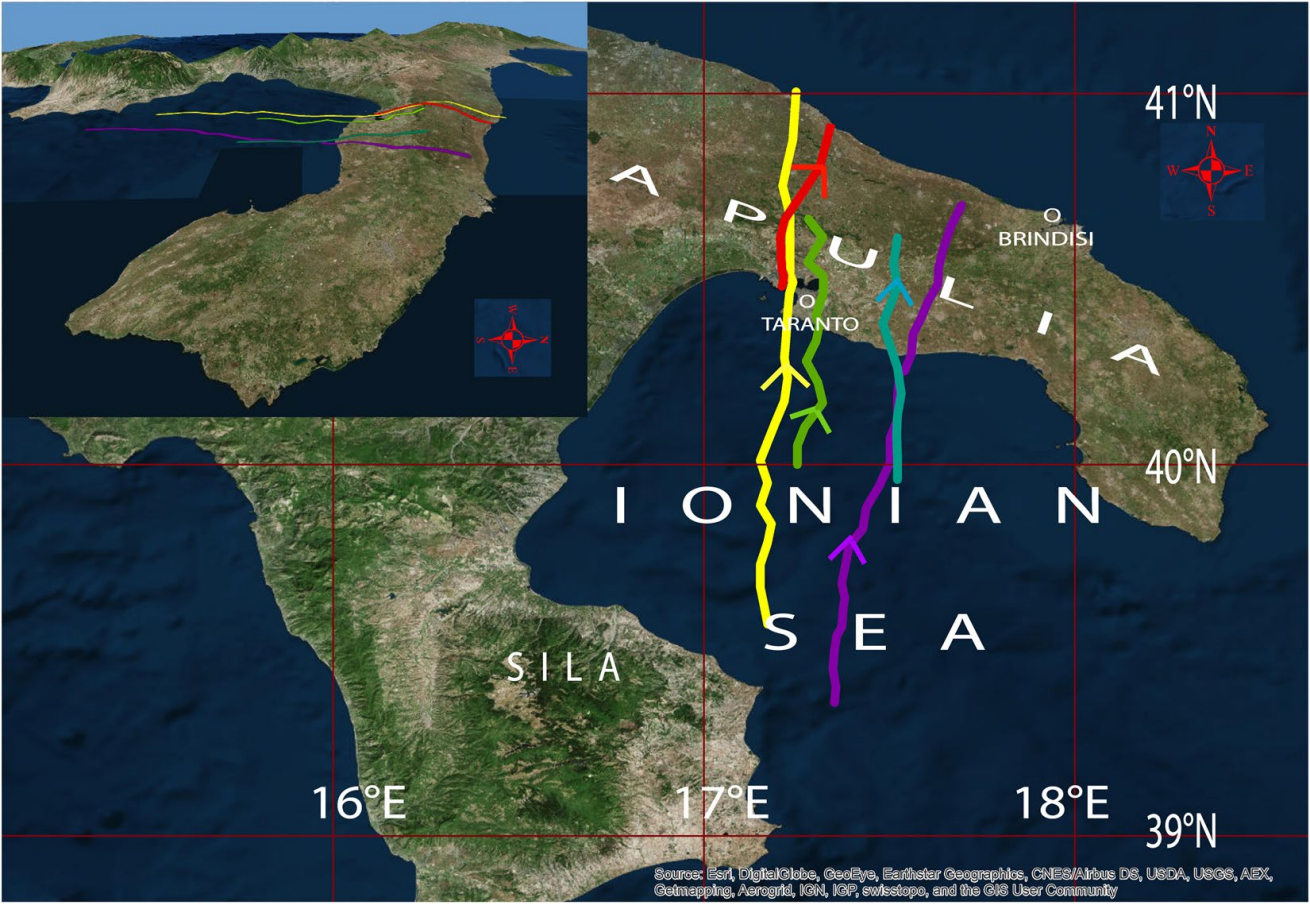
Tornado track and simulated supercell tracks. Observed tornado track (red), and supercell track simulated in the control run (yellow), in the run with SST increased by 1 K (purple), with SST increased by 0.5 K (cyan) and decreased by 0.5 K (green). (No supercell is simulated in the coldest run.) The simulated tracks are identified through the maximum vertical velocity at 500 hPa. The direction of displacement is shown with an arrow along the track. The geographic places mentioned in the text are also shown. The upper left inset shows the orography of the region (the vertical scale is magnified 15 times). (The figure has been generated with ESRI-ArcGIS, version 10.0, http://www.esri.com/arcgis/about-arcgis).

The exact track of the tornado over land^33^ is shown here in Fig. 1 (red line). The “proximity” sounding in Brindisi (70 km far from Taranto) at 12:00 UTC, 2 hours after the landfall, documented the extraordinary low-level wind shear (wind speed changed from 6 m s^−1^ at 10 m height to 28 m s^−1^ at 686 m height) responsible for the supercell rotation^32^.

The radar reflectivity images^32^ suggest that the orography south-southwest of Taranto played a key role in the development of the supercell: a line of convective cells was triggered by Sila mountains (top height of about 2000 m), then moved downstream and approached the coast near Taranto^31,32^. Motivated by the need for a better understanding of the dynamics of the event, numerical simulations were performed using the Weather Research and Forecasting (WRF) model^34^, implemented with 3 one-way nested grids, with horizontal spacing of 9, 3, and 1 km respectively^31^. This configuration is too coarse to simulate the tornado but can reproduce the supercell spawning it. A “control” simulation, using the ECMWF analysis/forecasts initialized at 00:00 UTC, 27 November 2012 as initial/boundary conditions (horizontal resolution of about 16 km) and lasting 36 hours, was able to simulate properly the evolution of the supercell, both in timing and track. Numerical simulations confirm the central role of Sila mountains in triggering convection and that both the increasing instability, due to the advection of high-*θ*_*e*_ (equivalent potential temperature) low-level air and cold mid-tropospheric air, and the intensification of deep- and low-level wind shear provided an environment favorable to supercell convection^31^.

### Sensitivity simulations

**Figure 2:**
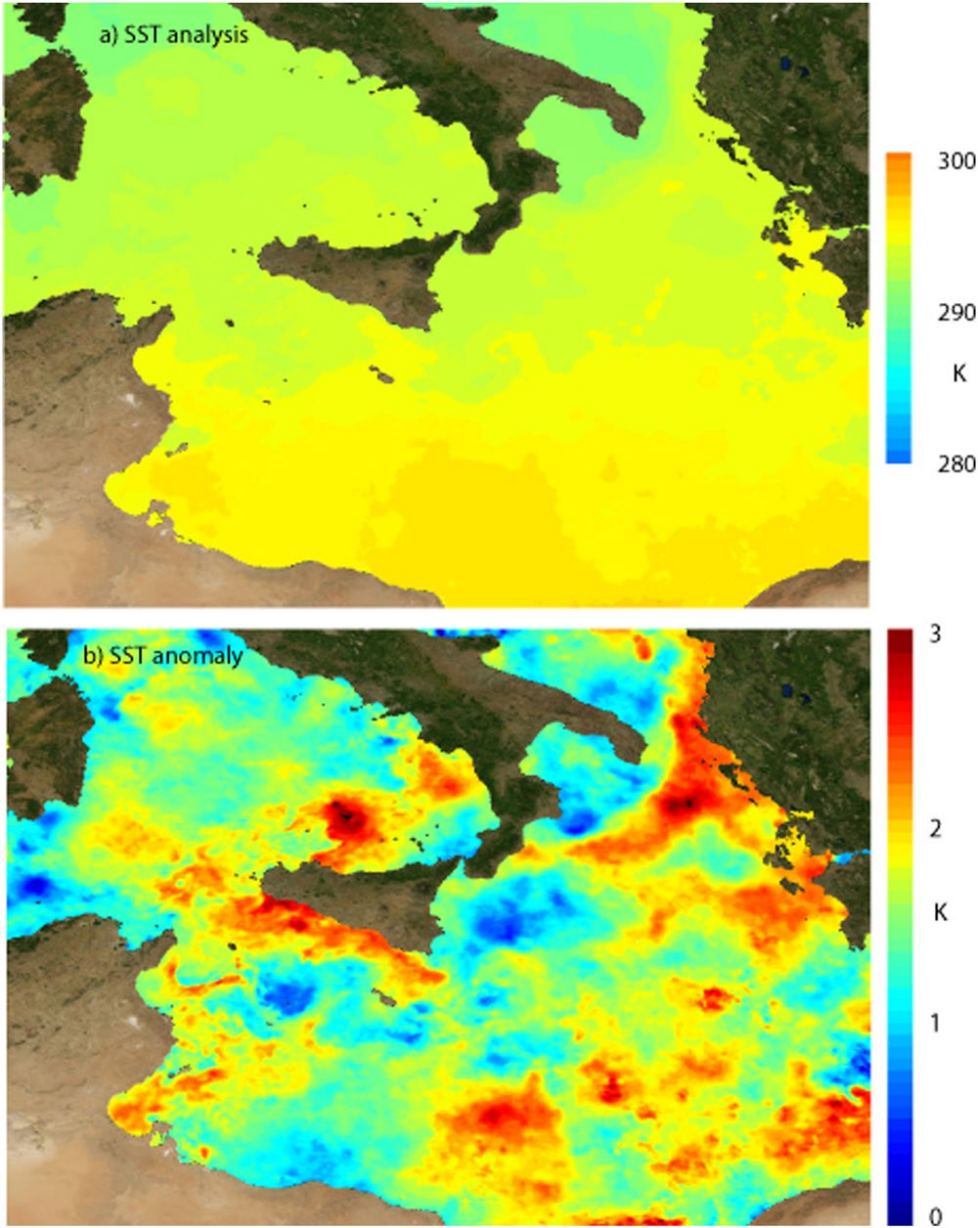
SST analysis and anomaly. Mediterranean Sea Ultra High Resolution Sea Surface Temperature analysis (**a**, top) and positive anomaly – with respect to the climatology 1985–2005 - (**b**, bottom) on 28 November 2012. The daily maps are gap-free at 0.01° × 0.01 ° horizontal resolution over the Mediterranean Sea^50^. The data are obtained from infrared measurements collected by satellite radiometers and statistical interpolation. (The figure has been created on-the-fly, using the E.U. Copernicus Marine Service Information, at the webportal: http://marine.copernicus.eu/services-portfolio/access-to-products/?option=com_csw&view=details&product_id=SST_MED_SST_L4_NRT_OBSERVATIONS_010_004).

We hypothesize that modifications in SST may affect the supercell intensity. SST values in the simulation (extracted from the ECMWF analysis at the initial time) are increased/decreased uniformly all over the domain by 0.5 K and 1 K with respect to the control run, while the atmospheric fields in the initial and boundary conditions are kept fixed. Figure 2 shows the SST analysis and anomaly in the southern part of the central Mediterranean. Since the observed SST anomaly was around +1 K in the area where the supercell developed (2012 experienced the maximum Ionian SST during Fall in the years 1982–2012^35^), the proposed experiments provide an indication of the way the supercell could have changed in the case of a normal SST or of a warm SST anomaly of +2 K, i.e. the mean climate change prediction of a moderate-forcing (RCP6.0) and a high-forcing (RCP8.5) CMIP5 (the Coupled Model Inter comparison Project 5) scenario^36^ over the Ionian Sea in Fall at the end of the 21^st^ century^35^.

Model outputs are saved every 5 minutes to represent the detailed evolution. The planetary boundary layer (PBL) at initial time is not adjusted to the modified surface temperature, but it is the same as in the control run. However, about 34 h elapse between the starting time of the runs (at 00:00 UTC, 27 November 2012) and the time of the landfall (simulated in the control run at around 10:20 UTC, 28 November), thus during the lifetime of the supercell the PBL has already relaxed to the modified lower boundary condition. As a consequence, in the morning of 28 November, the low-level temperature in each sensitivity experiment is different from that in the control run, being the difference maximum near the surface (by approximately the difference in SST between the runs) and progressively reduced moving to higher altitude.

The impact of SST on the supercell development is analyzed by comparing the sensitivity simulations with the control run. An indication of the intensity of the supercell is provided by the 2–5 km updraft helicity *UH*, a diagnostic parameter designed for identifying rotation in simulated storms. *UH* is computed by taking the integral of the vertical vorticity $\zeta$ times the updraft-vertical velocity ${w}$ between 2 and 5 km:

1 $$UH\,=\,{\int }_{2\,km}^{5\,km}w\zeta dz$$

A typical threshold used to predict mesocyclones^37^ is *UH* = 50 m^2^ s^−2^, while *UH* = 100 m^2^ s^−2^ was found to most reliably predict tornadoes^38^.

**Figure 3:**
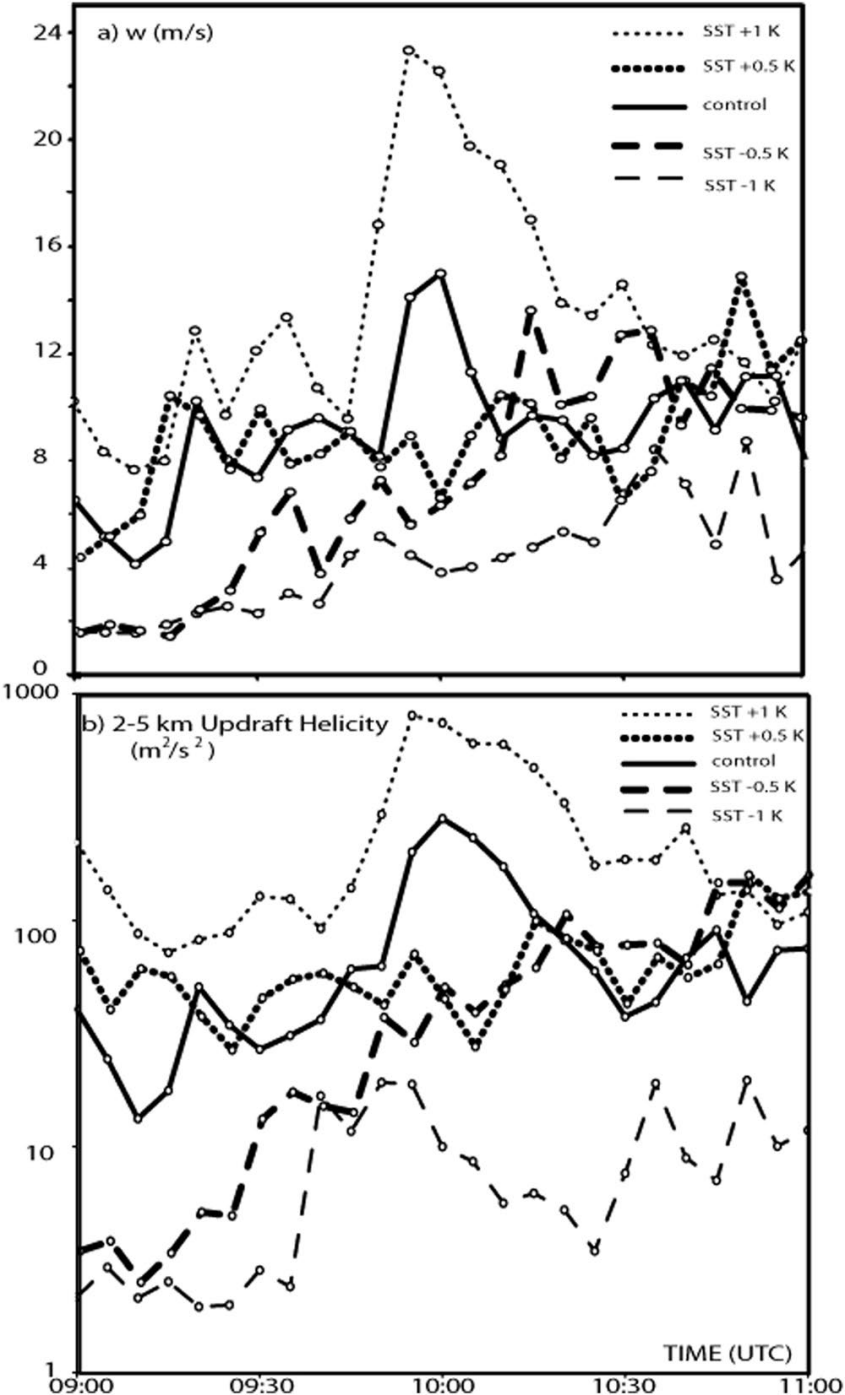
Updraft sensitivity: (**a**, top) Updraft vertical velocity at 600 hPa and (**b**, bottom) updraft helicity simulated in the run with SST decreased by 1 K (thin dashed line) and by 0.5 K (bolded dashed line), in the control run (bolded solid line), in the run with SST increased by 0.5 K (bolded dotted line) and by 1 K (thin dotted line) from 09:00 to 11:00 UTC, 28 November 2012. The values represent the maximum simulated in the window [lat = 39.7 °N, 40.7 °N; lon = 16.7 °E, 18.0 °E], inside the area where the supercell developed. In (**b**) the vertical scale is logarithmic.

In the control run, *UH* reaches peak values of 250 m^2^ s^−2^ just before landfall (Fig. 3). Within small variations of SST (+/− 0.5 K), the supercell still forms and the evolution appears similar to that in the control run (see Table 1), although convective activity appears more spread in the warmer simulation.

**Table 1:** .

FIELD	SST-1K	SST-0.5K	Control	SST+0.5K	SST+1K
600 hPa <w> (m/s)	4,8	7,5	9,3	9,8	13,6
600 hPa w_max_ (m/s)	10	13,5	15	16	23,5
450 hPa <w> (m/s)	4,1	9,1	11,6	11,8	15,6
450 hPa w_max_ (m/s)	9	17,5	23,5	20	27
UH_max_ (m^2^/s^2^)	53	165	280	160	810
<UH> (m^2^/s^2^)	15	53	75	76	229
MUCAPE (J/kg)	1180	1320	1450	1580	1940
SREH (m^2^/s^2^)	230	217	191	165	140

450 and 600 hPa updraft velocity maximum (w_max_) and its time and area average between 09:00 and 12:00 UTC (<w>), updraft helicity maximum (UH_max_) and its time and area average between 09:00 and 12:00 UTC (<UH>) (the area averages are performed in the window [lat = 39.7°N, 40.7°N; lon = 16.7°E, 18.0°E], where the supercell developed), *MUCAPE* and 0–3 km storm relative helicity (SREH) averaged in the period 09:30–10:00 UTC (around the time of the supercell formation) in the window [lat = 39.6 °N, 39.9 °N; lon = 17.1 °E, 17.7 °E], i.e. close to the area where the supercell originated.

However, when SST is modified by 1 K, the changes are dramatic and highly nonlinear. Near the time of the observed landfall, the simulated *UH* span two orders of magnitude for a SST variation of just 2 K. In the coldest run, only a limited peak of *UH* ≈ 20 m^2^ s^−2^ is simulated (some cells are triggered by the Sila mountains, but no supercell forms); in contrast, the case with the warmest SST produces a strong intensification of the updraft rotation in the supercell, since a peak of *UH* higher than 800 m^2^ s^−2^ is reached when the cell gets close to the coastline near Taranto (Fig. 3). In the latter case, the track is slightly shifted to the east compared to the control run (Fig. 1, purple line), thus the longer persistence of the cell over the sea may have also cooperated to its stronger intensification.

The difference in *UH* follows from changes in sea surface fluxes, in particular latent heat fluxes. Indeed, the lower troposphere is moistened and warmed with greater intensity for higher SST, thus the low-level profiles of temperature and humidity differ significantly among the experiments, affecting potential instability. As a consequence, the values of *MUCAPE* (Convective Available Potential Energy of the Most Unstable parcel^39^) around the time when the supercell developed, averaged in the area where it originated, range from 1180 J kg^−1^ for the coolest case to 1940 J kg^−1^ for the warmest case (Table 1): greater *CAPE* means stronger updrafts, hence more intense stretching of low-level environmental vorticity.

Figure 3a show that the maximum vertical velocity *w* at 600 hPa increases with SST. Table 1 shows that the range of variation within 0.5 °C around the SST of the control run is quite limited, while *w* is much greater in the warmest case and smaller in the coldest case. Similar results come out for other levels (e.g., *w* at 450 hPa, about the level where the uplift was found to be maximum, is shown in Table 1). Comparing *w* at different levels, one can note that *w* is lower at 450 hPa than at 600 hPa only in the coldest case, thus suggesting the presence of less vigorous and shallower convection in that run.

Finally, several instability indices used to diagnose severe convection were analyzed. Although most parameters show conditions slightly more favorable to severe convection for higher SST (not shown), only *CAPE* identifies a significant change of the environmental characteristics (Table 1). While an increase in CAPE by around 200 J kg^−1^ was recently simulated for a 2 K increase in the Mediterranean SST^40^, in the present study Table 1 shows that for the analyzed supercell the same change in SST would produce greater modifications in CAPE and, consequently, in the updraft velocity.

In contrast, the 0–3 km storm relative helicity

2 $${\rm{SREH}}\,=\,{\int }_{0\,km}^{3\,km}({\boldsymbol{v}}-{\boldsymbol{c}})\cdot {\boldsymbol{\omega }}dz$$

(where: ***v*** is the horizontal wind vector, **c** is the storm motion vector, ${\boldsymbol{\omega }}$ is the horizontal vorticity vector associated with the vertical wind shear), which is a measure of the potential for cyclonic updraft rotation, slightly decreases for increasing SST. This change may contribute to explain the non-monotonic variation of *UH*_*max*_ between the control run and the simulation with SST increased by 0.5 K (Table 1).

## Discussion

In the last years, due to the critical impacts of extreme events on territories, ecosystems and humans, a strong interest for their attribution has raised in order to understand how much the changes in their features can be due to the anthropogenic factors manifested in the climate change. Modelling studies are usually oriented to understand if the number of extreme events increases with global warming, but the topic of their intensity is only partially addressed^41,42^. Of course, attention is paid mainly to large-scale processes and phenomena, due to difficulties in modelling severe localized weather. In this framework, the work presented here represents a first attempt at investigating how an increase in SST, predicted for the next decades, may affect the thermodynamics of a tornadic supercell and lead to stronger effects/impacts.

The analysis of this specific event suggests that, in similar environmental conditions, a warm SST anomaly, due to a temporary and local fluctuation in the field or induced by a general SST warming associated with climate change, may favor supercell formation and intensification over the Mediterranean. Considering that the supercell developed over the Ionian Sea, which was warmer than average by about 1 K, one can speculate that no supercell development would have occurred over a normal SST, while an even warmer anomaly would have drastically increased the intensity of the supercell (and, possibly, of the associated tornado) in a nonlinear manner.

However, this does not necessarily imply that the frequency of these events should increase in future climate scenarios, considering that the present study only deals with changes in thermodynamics; dynamical modifications in weather circulation patterns, important as well to explain climatological changes in extreme weather events^43^, are not analyzed here. (Incidentally, some studies agree that the effect of climate change on Mediterranean tropical-like cyclones is to decrease their frequency, although they would develop over a warmer SST^44^). Nonetheless, our analysis is consistent with climate change studies, which predict an increase of tornado activity in global warming scenarios due to the increasing CAPE (which more than compensates for the predicted decrease in SREH). Idealized simulations are actually in progress to better understand the role of the forcing mechanisms in the development of the analyzed supercell.

## Methods

### Numerical setup

The Weather Research and Forecasting (WRF) model, version ARW-3.5.1 (ref.^33^) is used for the numerical simulations of the case study. WRF is a limited area model, which solves the fully compressible, nonhydrostatic primitive equations. Forty terrain-following vertical levels are used, more closely spaced near the ground to better represent the boundary layer (their vertical distance ranges from 58 m in the boundary layer to 600 m).

Three one-way-nested domains are implemented. The outer grid covers the central part of the southern Mediterranean (210 × 150 grid points, dx = 9 km), the intermediate grid represents southern Italy, part of Greece and Albania (271 × 193 points, dx = 3 km); the inner domain is centered over the Ionian regions of southern Italy (211 × 271 points, dx = 1 km).

The following parameterization schemes are used: the Thompson microphysics^45^; the longwave radiation Rapid Radiative Transfer Model (RRTM)^46^; the Dudhia shortwave radiation^47^; the land-surface unified Noah model^48^; the Mellor–Yamada–Janjic planetary boundary layer^49^. Cumulus convection is switched off in all domains.

The “control” simulation uses the ECMWF analysis (forecasts) initialized at 00:00 UTC, 27 November 2012 as initial (boundary) conditions and lasts 36 hours. The sensitivity runs use the same setup, apart from the modified sea surface temperature.

### Data availability

Simulation outputs and data to reproduce the numerical experiments are available on request.
